# 

**Published:** 1841-02

**Authors:** 


### CONTENTS

### OF NO. II.

### ORIGINAL COMMUNICATIONS.

### ESSAYS AND CASES.

Art. I.—Desultory Notes of a Medical Traveller. In a letter to one of the Editors, dated Staunton, Va., July 7th, - 81

Art. II.—A Case of pregnancy attended by a remarkable discharge from the Uterus, followed by a safe Delivery. By S. E. Leonard, M. D., of New Albany, Ia. - - - 87

Art. III.—A Case of Traumatic Tetanus successfully Treated. By E. F. Wilson, M. D., of Louisville, Ky. - - 91

Art. IV.—A Case of Congenital Deafness, relieved by an operation performed by the patient on himself. Reported for this Journal by S. M. Knapp, Esq., of Winchester, Ill. - 95

Art. V.—A Case of Peritonitis, consequent upon Bilious Fever, terminating in a copious secretion of pus into the cavity of the Abdomen. By A. H. Buchanan, M. D., of Columbia, Tenn. - - - - - - - - 101

Art. VI.—Observations on a peculiar form of Bilious Fever. By John Hardin, M, D., of Greensburg, Ky. - - 1[illegible]

### REVIEWS.

Art. VII.—Outlines of the Institutes of Medicine; founded on the Philosophy of the Human Economy, in health and disease. In three parts. By Joseph A. Gallup, M. D. - - - - - - - - - - 113

### SELECTIONS FROM AMERICAN AND FOREIGN JOURNALS.

On pain of the back in Remittent Fever. - - - - - - 141
External Application of the Oil of Ergot. - - - - - 142
Solution of Bi-chloride of Mercury in Itching. - - . - 143
On the Causes of Scrofulous Diseases. - - - - - - - 143
Treatment of local Paralysis by Phosphorus. - - - - 144
New Operation for Prolapsus Ani. By M. Robert. - - - 145
New facts with regard to Strangulated Hernia. By M. Laugier. - 146
Treatment of Diseases of the Testicle by Compression. - - 147
Case of Phlebitis Successfully Treated. By George Bell, M. D. - 149

### ORIGINAL INTELLIGENCE.

Medical Convention of Kentucky. - - - - - - - 157
Bilious and Congestive Fever. By A. M. Keller, M. D., of Alabama. - - - - - - - - - - 159

